# Effects of Taurine and Vitamin C on the Improvement of Antioxidant Capacity, Immunity and Hypoxia Tolerance in Gibel Carp (*Carrassius auratus gibeilo*)

**DOI:** 10.3390/antiox13101169

**Published:** 2024-09-26

**Authors:** Leimin Zhang, Lu Zhang, Hualiang Liang, Dongyu Huang, Mingchun Ren

**Affiliations:** 1 Wuxi Fisheries College, Nanjing Agricultural University, Wuxi 214081, China; 2 Tongwei Agricultural Development Co., Ltd., Key Laboratory of Nutrition and Healthy Culture of Aquatic, Livestock and Poultry, Ministry of Agriculture and Rural Affairs, Healthy Aquaculture Key Laboratory of Sichuan Province, Chengdu 610093, China; 3 Key Laboratory of Integrated Rice-Fish Farming Ecology, Ministry of Agriculture and Rural Affairs, Freshwater Fisheries Research Center, Chinese Academy of Fishery Sciences, Wuxi 214081, China

**Keywords:** gibel carp (*Carrassius auratus gibeilo*), hypoxia, taurine, vitamin C, survival and body condition, antioxidant capacity, dietary supplementation

## Abstract

To investigate the effects of taurine and vitamin C on gibel carp (*Carrassius auratus gibeilo*), fish (41.85 ± 0.03 g) were fed three diets with 0% taurine + 0% vitamin C (D0), 0.1% taurine + 0% vitamin C (D1), and 0.1% taurine + 0.1% vitamin C (D2) for 8 weeks. Then 12-hour hypoxic stress test was conducted. The results showed that weight gain rate (WGR), specific growth rate (SGR), and sustained swimming time (SST) were significantly increased in the D2. CAT, SOD, T-AOC, and GSH were increased. GSH-Px and *il-6* were decreased in D1 and D2. In hypoxia, CAT and T-AOC were decreased, while GSH, *sod*, and *nrf2* were the highest in D1. Compared to normoxia, GSH-Px was increased, while SOD and MDA were decreased. *Il-10* and *nf-κb* were increased. *Vegf*, *epo*, and *ho-1* were increased and they all were higher than that in normoxia. The number of gill cell mitochondria and survival rate (SR) of gibel carp had an increasing trend but no significant difference among groups. In conclusion, taurine with vitamin C improved the growth and SST of gibel carp, and taurine and taurine with vitamin C improved antioxidant capacity, immunity, and hypoxia tolerance.

## 1. Introduction

Hypoxia is one of the main environmental factors limiting the healthy development of aquaculture [1,2,3]. Dissolved oxygen (DO) is necessary for the survival of aquatic animals. When the DO concentration is less than 2 mg/L, the aquatic environment is hypoxic. This can lead to various negative effects on aquatic animals, such as reduced food intake, abnormal movement, and even death [4,5]. Various attempts have been made to mitigate the effects of hypoxia on the health of fish. Studies have shown that the use of feed additives to improve fish tolerance is effective. For example, the appropriate addition of arginine to the feed can be helpful to improve the survival of the Indian major carp (*Cirrhinus mrigala*) under hypoxia [6]. The appropriate levels of γ-aminobutyric acid (GABA) in the feed can modulate the HIF-1 signaling pathway and blood biochemical levels of the Indian major carp to improve its survival under hypoxia [7]. The appropriate addition of mulberry leaf extract can reduce excess reactive oxygen species (ROS) in gibel carp (*Carrassius auratus gibeilo*), reduce oxidative damage, and improve their ability to tolerate hypoxia [8]. Therefore, food supplements are a viable method to alleviate the negative effects of hypoxic stress with the help of food.

Taurine is an organic acid that was originally isolated from the bile of bulls and is also known as sulphonic acid or sulphonic amino acid [9]. As a non-protein amino acid, taurine is not involved in protein synthesis and does not provide energy; however, it has been found in large amounts in tissues, accounting for 30–50% of total amino acids, and is an important free amino acid in the tissues of the body [10,11]. In recent years, the nutritional and physiological functions of taurine in aquatic animals and its use in feed have attracted much attention. The results of related studies have shown that the addition of taurine to feed can improve fish growth [12,13,14,15]. Other studies have shown that taurine can improve the ability of animals to tolerate hypoxia, prevent hypoxia-induced death of hepatocytes in rats [16], and increase the hypoxia tolerance of juvenile grass carp (*Ctenopharyngodon idellus*) [17]. Therefore, taurine is a beneficial additive for aquatic animals.

Vitamin C, also known as ascorbic acid, is a water-soluble antioxidant that can reduce ROS and protect cells from oxidative damage. It is an essential nutrient for animal growth and metabolism and can also improve fish growth, boost immunity and antioxidant capacity, and relieve stress [18,19]. Related studies have shown that the adequate addition of vitamin C to feed can improve the growth performance of coho salmon (*Oncorhynchus kisutch*) [20], silver carp (*Hypophthalmichthys molitrix*) [21], and tiger pufferfish (*Takifugu rubripes*) [19], while adequate levels of vitamin C can also boost the immunity of fish [22,23,24]. Using antioxidant enzymes as indicators, an appropriate amount of vitamin C can be used to increase the antioxidant capacity of fish [21,25,26]. An adequate supply of vitamin C as a coenzyme is beneficial to the growth and physical health of fish.

The gibel carp, *Carrassius auratus gibeilo*, is an important freshwater aquaculture species that plays a prominent role in meeting the human need for proteins and aquatic products [27]. However, climatic factors and intensive aquaculture may also expose gibel carp to the dangers of hypoxic stress, affecting its growth and production. Previous studies have shown that taurine can be used as a feed supplement to effectively improve the growth and hypoxia tolerance of some fish; however, a corresponding study on gibel carp has not yet been conducted. In addition, it is worth exploring whether hypoxic tolerance can be further improved by the combined effects of taurine and vitamin C. Therefore, this study was designed to investigate the effect of taurine and the combined effect of taurine and vitamin C on the growth performance, antioxidant capacity, immunity, and hypoxia tolerance of gibel carp in order to improve the growth and stress resistance of fish and promote the stable development of aquaculture.

## 2. Materials and Methods

### 2.1. Diet Preparation

Based on previous studies [13,28], we designed three experimental diets by adding different amounts of taurine and vitamin C to the normal diet: 0% taurine + 0% vitamin C (D0, control group), 0.1% taurine + 0% vitamin C (D1), and 0.1% taurine + 0.1% vitamin C (D2). Table 1 lists the normal feed composition. The amino acid balancing method was based on our previous study [29]. Fish, chicken, soybean, cottonseed, and rapeseed meals were used as the main protein sources. Soybean oil was the main lipid source. The feed production process includes crushing, sieving, weighing, mixing, and pelleting [30]. The ingredients were crushed, sieved, proportioned, mixed with water and oil according to the feed formulation, pelleted in a pelletizing machine (F-26(II), South China University of Technology, Guangzhou, China), and dried. The feed was frozen at −20 °C for further use.

### 2.2. Experimental Procedure

Fish were obtained from the Freshwater Fisheries Research Center. At the beginning of the feeding trial, the fish were fasted for 24 h and then weighed. A total of 180 healthy gibel carp (41.85 ± 0.03 g) were randomly divided into three experimental groups with three replicates each, resulting in a total of nine cages. Fish were fed twice daily based on apparent satiety (6:30 and 16:30). Fish mortality was recorded daily. Feeding trials were conducted in 1 m × 1 m × 1 m floating cages and maintained for 8 weeks. Water quality was continuously monitored, and the water temperature was 28 ± 2 °C. DO was maintained at ≥6.5 mg/L, and total ammonia nitrogen was below 0.1 mg/L throughout the feeding process. Aquaculture experiments were conducted at the Nanquan Aquaculture Basement of the Freshwater Fisheries Research Center (Wuxi, China).

### 2.3. Sample Collection

After the feeding trial, fish were fasted for 24 h before sampling. Fish in all cages were weighed to obtain data on growth performance. Two randomly selected fish from each cage were used to collect liver tissue samples. Liver samples were collected and temporarily stored in a tank of liquid nitrogen at −196 °C. After sampling, all liver samples were stored at −80 °C for subsequent gene expression analysis and determination of antioxidant enzyme activity. Two additional fish were randomly selected from each cage and stored at −20 °C for whole-body composition analysis. A SY28060 swimming flume (Loligo Systems, Viborg, Denmark) was used to test the swimming ability of gibel carp. Specific measurements were based on previous studies [31]. One fish was placed in the flume test area. The flow velocity was set at 5 cm/s to acclimatize the fish, and the flow velocity of the water was gradually increased to 30 cm/s. At the same time, the time between the fish swimming against the current and the point at which the fish swam with the current to the wall of the flume and stopped moving was recorded as the sustained swimming time (SST).

The remaining fish were safely transferred to a recirculating water culture system (300 L per tank) for hypoxia experiments. Before starting hypoxic stress, the water surface in the tanks was set high enough to allow the fish to swim freely, and the water surface was covered with a transparent suspension film to prevent oxygen exchange between air and water. The DO content of the water was monitored during the experiment using a portable dissolved oxygen meter. Stress was triggered when the DO content in the water dropped to 0.8 ± 0.1 mg/L. The stress period was 12 h, during which the fish in each tank were observed and recorded. Twelve hours later, the SR of the fish was noted. During the process, the water temperature was approximately 19.5 °C. Liver tissue was collected from six of the remaining fish for analysis of gene expression and antioxidant enzyme activity, and gill tissue was collected for transmission electron microscopy.

### 2.4. Chemical Analysis

The moisture, crude protein, crude lipid, and ash contents (%) of the experimental feed and whole fish body were measured using standard methods with three replicates per group [32]. The samples were first dried in an oven at 105 °C to determine the moisture content. Subsequently, crude protein content was measured using the Kjeldahl nitrogen determination method on an automatic instrument (Haineng K1100, Jinan Haineng Instrument Co., Ltd., Jinan, China). Crude lipid content was analyzed by the Soxhlet extraction method in an automatic fat analyzer (Haineng SOX606, Jinan Haineng Instrument Co., Ltd., China) and samples were calcined at 560 °C for 6 h in a Muffle furnace (XL-2A, Hangzhou Zhuochi Instrument Co., Ltd., Hangzhou, China) to obtain ash content data. Activities of antioxidant enzymes such as catalase (CAT) (U/mgprot), superoxide dismutase (SOD) (U/mgprot), glutathione peroxidase (GSH-Px) (U/mgprot), total antioxidant capacity (T-AOC) (mmol/gprot), glutathione (GSH) (μmol/gprot), and malondialdehyde (MDA) (nmol/mgprot) were measured using corresponding kits provided by Nanjing Jiancheng Bioengineering Institute (Nanjing, China) following previous studies. The expertise of the measurement methods is widely recognized by universities and research institutes [33,34,35,36]. The main instrument used was the spectrophotometer (Thermo Fisher Multiskan GO, Shanghai, China). The succinct methods of determining antioxidant enzyme activities as well as MDA are shown in Table 2. The specific methods for determination were as follows: All operations were performed in strict accordance with the instructions of the kits. (1) CAT was measured using the kit model A007-1-1 by the ammonium molybdenum acid method. Each milligram of protein in the sample corresponds to 1 µmol of H_2_O_2_ decomposed by CAT per second as one unit of activity (U). (2) T-AOC was determined using the kit A015-2-1 by the ABTS method. The principle is that ABTS is oxidized to green ABTS+ in the presence of a suitable oxidant. Antioxidants inhibit the production of ABTS+. The total antioxidant capacity of a sample can be determined and calculated by measuring the absorbance of ABTS+ at 405 nm or 734 nm, and Trolox is used as a reference for the total antioxidant capacity of antioxidants. (3) The activity of SOD is determined using kit model A001-3-2 with the WST-1 method, and the meaning of the result is that one unit of SOD activity (U) is the amount of enzyme required to inhibit 50% of the oxidation rate per reactive solution and per milligram of protein in 1 mL of reactive solution. (4) The TBA method was applied to determine MDA using the kit model A003-1-2. The principle is that MDA in the degradation products of lipid peroxidation can condense with thiobarbituric acid (TBA) to form a red product with a maximum absorption peak at 532 nm. Therefore, the results of the determination at this wavelength can reflect the content of MDA. (5) The kit model A006-2-1 using the microplate method was used for the determination of GSH. The principle of the determination is that GSH can react with dithiobisnitrobenzoic acid to produce a yellow compound, which can be used for colorimetric quantitative determination of the content of GSH under the condition of 405 nm wavelength. (6) The activity of GSH-Px is measured using the kit A005-1-2 by the colorimetric method. A unit of enzyme activity (U) is defined as one unit of enzyme activity (U) per milligram of protein per minute at 37 °C, minus the effect of the nonenzymatic reaction, which reduces the concentration of GSH in the reaction system by 1 μmol/L. The calculation of all the above indexes needs the protein content in liver tissue, so we use the kit model A045-2-2, applying the Caulmers Brilliant Blue method to determine the protein content. The principle is that the protein molecule has the -NH^3+^ group. When the brownish-red Caulmers Brilliant Blue colorant is added to the protein standard solution or the sample, the anion on the Caulmers Brilliant Blue dye combines with the protein -NH3+, making the solution turn blue. The solution turns blue, and the protein content can be calculated by measuring the absorbance. Gill cell sections were prepared by Wuhan Servicebio Technology Co., Ltd. (Wuhan, China).

### 2.5. RNA Extraction and Real-Time PCR Analysis

RNA was extracted from the liver tissue of gibel carp using an RNA extraction reagent (Vazyme, Nanjing, China). The concentration and quality of RNA were determined using a NanoDrop 2000 spectrophotometer. The concentration of the RNA samples was adjusted to 60 ng/μL, with A260/A280 ranging between 1.8 and 2.0. qPCR was performed using the One-Step SYBR Prime Script TM PLUS RT-PCR kit (Takara, Dalian, China) on a CFX96 Touch (Bio-Rad, Hercules, CA, USA). The qPCR reactions were programmed as follows: reverse transcription at 42 °C for 5 min, pre-denaturation at 95 °C for 10 s, then denaturation at 95 °C for 5 s and 60 °C (annealing temperature) for 30 s for 40 cycles. The melting curves were analyzed by increasing the temperature from 65 °C to 95 °C while observing fluorescence. Beta-actin (*β-actin*), which has high and stable expression, was chosen as the internal reference gene, and no significant difference was detected. The primer sequences used are listed in Table 3. Some of the primer sequences were obtained from previous research, and the remaining primers were designed online using Primer Premier 6.0. Sangon Biotech (Shanghai) Co., Ltd. (Shanghai, China) helped to synthesize the primers. The standard curve method was used to calculate the relative expression of genes [33].

### 2.6. Statistical Analysis

The data were subjected to normality and homogeneity tests before any statistical analysis. The statistical software SPSS 25.0 was used to analyze all data. A one-way ANOVA (Duncan’s test) was used to evaluate the effects of the addition of taurine and vitamin C on growth performance, whole-body composition, SST in normoxia, SR, and the number of cellular mitochondria in hypoxia. The effects of DO and the addition of taurine and vitamin C, their interaction on the liver genes, and the antioxidant enzyme activities of gibel carp were evaluated using a two-way ANOVA. When a significant interaction between the main effects of the variables was observed, the main effects of taurine and vitamin C were evaluated using a one-way ANOVA (Duncan’s test), and those of DO were evaluated using an independent samples *t*-test. The results are presented as means with standard deviations. Statistical significance was set at *p* < 0.05.

## 3. Results

### 3.1. Growth Performance

The growth performance-related indices and feed conversion ratio (FCR) are listed in Table 4. With the addition of taurine and vitamin C, final body weight (FBW), specific growth rate (SGR), and weight gain rate (WGR) showed an increasing trend. Among all groups, WGR and SGR were highest in the D2 group, and both were higher than those in the D0 group (*p* < 0.05), and the survival rate (SR) of all groups was 100%. The D2 group showed an increasing trend and the highest FCR, but no significant difference was observed (*p* > 0.05).

### 3.2. Whole-Body Composition

The whole-body composition of gibel carp is shown in Table 4. Moisture content was lower in both the D1 and D2 groups than in the D0 group. Crude protein content was lower in the D1 group than in the D0 group, whereas it was highest in the D2 group. The crude lipid content showed a decreasing trend and reached the lowest value in the D2 group, whereas the ash content showed an increasing trend and reached the highest value in the D2 group. None of the above body composition indices showed a significant difference between the groups (*p* > 0.05).

### 3.3. Swimming Ability

Table 4 shows the swimming ability of the different groups of gibel carp. No significant difference in SST was found between groups D1 and D0, while the SST of group D2 was significantly longer than those of the other two groups (*p* > 0.05).

### 3.4. Antioxidant Capacity of the Liver (Nrf2 Signaling Pathway)

Results of the antioxidant enzyme activities in gibel carp liver under normoxic and hypoxic environments are shown in Table 5. CAT, T-AOC, SOD, and GSH levels tended to increase with the addition of taurine and taurine with vitamin C in a normoxic environment, and the differences were significant compared with the D0 group (*p* < 0.05). GSH-Px tended to decrease (*p* < 0.05), and no significant difference was observed in MDA. In a hypoxic environment, CAT and T-AOC showed a decreasing trend, and GSH showed an increasing, and then decreasing trend, and the maximum value was observed in the D1 group (*p* < 0.05). The differences were not significant in SOD, MDA, and GSH-Px levels between the groups. The results of the two-way ANOVA showed that DO has a significant effect on SOD, MDA, and GSH-Px. Compared with normoxia, SOD and MDA were significantly decreased, and GSH-Px was significantly increased in hypoxia (*p* < 0.05), while no significant difference was found in the other indices. The addition of taurine and taurine with vitamin C significantly affected the T-AOC and GSH levels. The interaction of DO and the addition of taurine or taurine with vitamin C had significant effects on CAT, T-AOC, SOD, and GSH-Px (*p* < 0.05).

**Figure 1:**
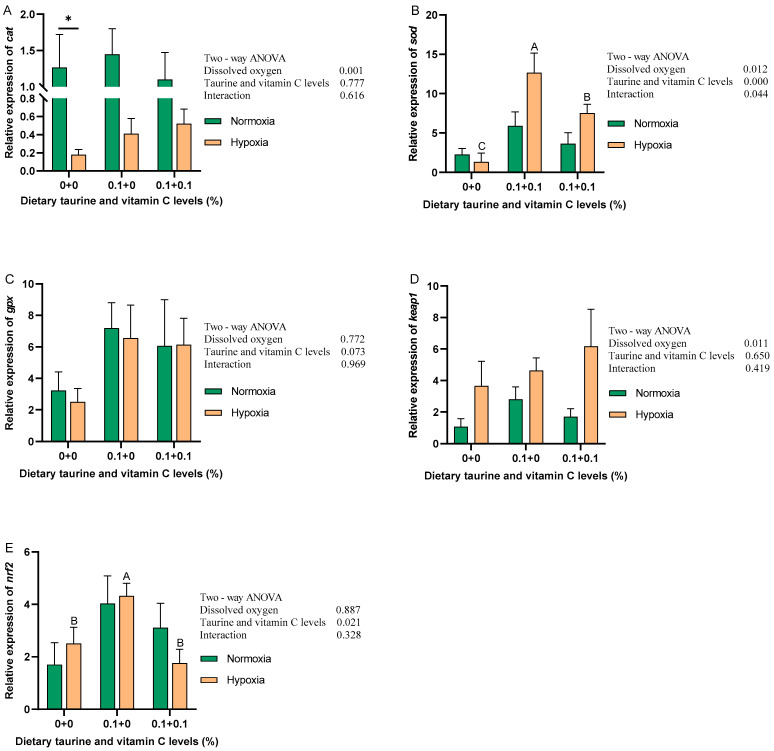
Relative expression levels of mRNA of antioxidant-related genes of gibel carp fed graded dietary of taurine and vitamin C in normoxia for 8 weeks and hypoxia for 12 h. Data with different superscript letters are significantly different (*p* < 0.05); the hypoxic data were labeled with an upper case “ABC”, “*” indicates a significant difference (*p* < 0.05) between values of 0.8 mg/L and 6.5 mg/L dissolved oxygen as determined by *t*-test. (**A**) *cat*, catalase; (**B**) *sod*, superoxide dismutase; (**C**) *gpx*, glutathione peroxidase; (**D**) *keap1*, recombinant kelch-like ECH-associated protein 1; (**E**) *nrf2*, nuclear factor erythroid 2-related factor 2.

Figure 1 shows the expression levels of antioxidant-related genes in gibel carp liver under normoxic and hypoxic environments. From Figure 1, we observe that in the normoxic environment, after adding taurine and taurine with vitamin C, *sod*, *gpx*, and *nrf2* had an increasing trend, but none of them had significant differences (*p* > 0.05). *cat* and *keap1* had no significant trend. In the hypoxic environment, the expression of *sod* and *nrf2* increased and then decreased after the addition of taurine and taurine with vitamin C (*p* < 0.05). The expression levels of *cat*, *gpx*, and *keap1* were positively associated with the addition of taurine and taurine with vitamin C, but none were significantly different (*p* > 0.05). The results of the two-way ANOVA showed that DO significantly affected the expression of *cat*, *sod*, and *keap1*, while the addition of taurine and taurine with vitamin C significantly affected *sod* and *nrf2*. The expression of *sod* was significantly affected by the interaction between DO and the addition of taurine and taurine with vitamin C.

### 3.5. Immune Response of the Liver (NF-κB Signaling Pathway)

**Figure 2:**
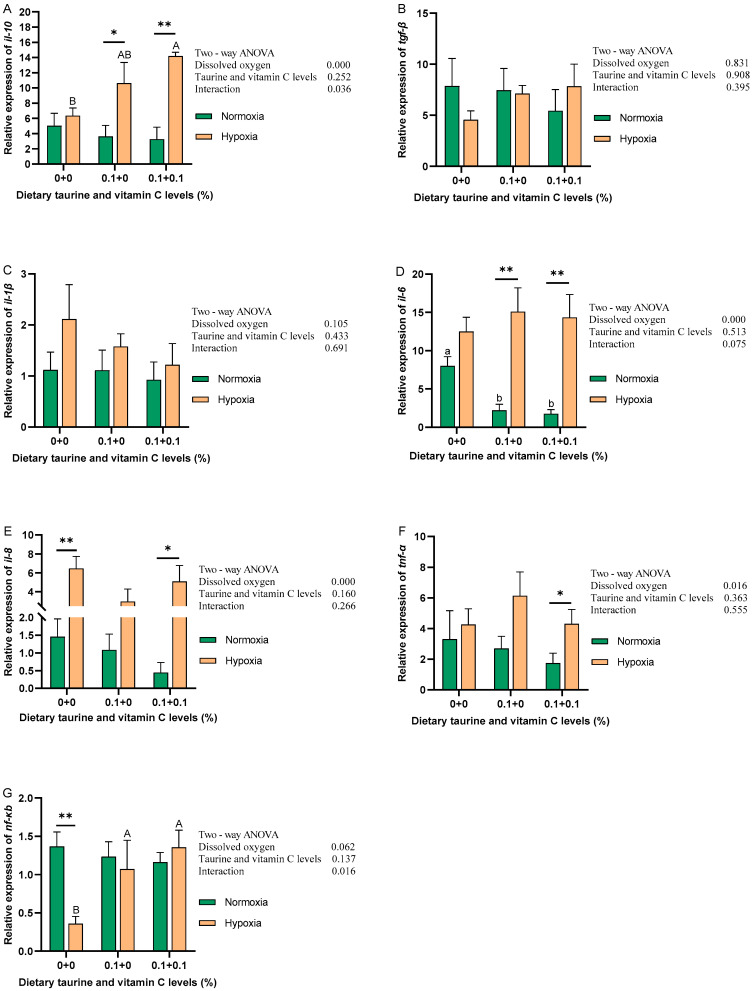
Relative expression levels of mRNA of hepatic inflammation-associated genes of gibel carp fed graded dietary of taurine and vitamin C in normoxia for 8 weeks and hypoxia for 12 h. Data with different superscript letters are significantly different (*p* < 0.05); the normoxic data were labeled with a lower case “ab” and the hypoxic data were labeled with an upper case “AB”, “*” indicates a significant difference (*p* < 0.05) and “**” indicates a significant difference (*p* < 0.01) between values of 0.8 mg/L and 6.5 mg/L dissolved oxygen as determined by *t*-test. (**A**) *il-10*, interleukin-10; (**B**) *tgf-β*, transforming growth factor-β; (**C**) *il-1β*, interleukin-1β; (**D**) *il-6*, interleukin-6; (**E**) *il-8*, interleukin-8; (**F**) *tnf-α*, tumor necrosis factor-α; (**G**) *nf-κb*, nuclear factor kappa-β.

The expression levels of immune-related genes in gibel carp liver under normoxic and hypoxic environments are shown in Figure 2. In the normoxic environment, all inflammatory factors showed a decreasing trend, and the expression of *il-6* in the D1 and D2 groups was significantly lower compared to the D0 group (*p* < 0.05); the expressions of the rest of the genes were not significantly different among groups. In hypoxia, as the additions of taurine and taurine with vitamin C, an increasing trend in the expression of *il-10*, *tgf-β*, and *nf-κb* was observed in gibel carp liver, among which the changes in *il-10* and *nf-κb* were significant (*p* < 0.05). There were decreasing trends in the expressions of *il-1β* and *il-8*, but the differences were not significant. The remaining genes showed no significant change in trend. The results of a two-way ANOVA showed that DO could significantly affect the expression of *il-10*, *il-6*, *il-8*, and *tnf-α* (*p* < 0.05). The additions of taurine and taurine with vitamin C did not significantly affect the expression of each gene; *il-10* and *nf-κb* were significantly affected by the interaction of DO and the additions of taurine and taurine with vitamin C.

### 3.6. Hypoxia Signaling Pathway (HIF-1 Signaling Pathway)

**Figure 3:**
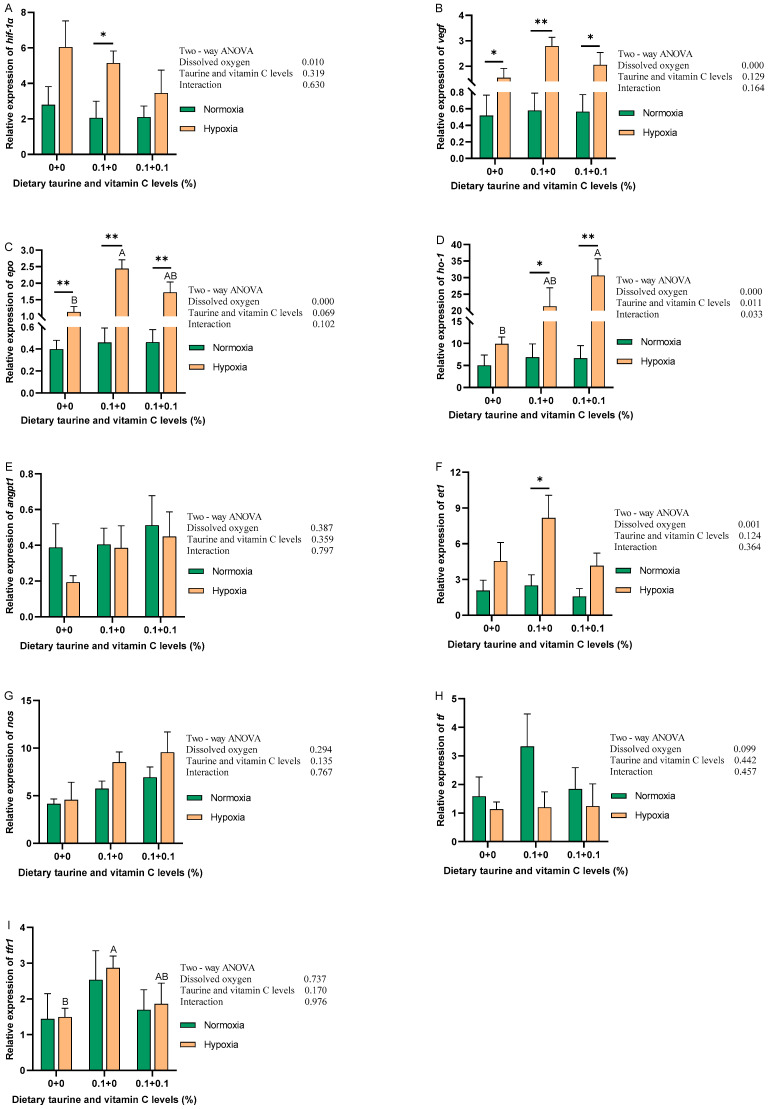
Relative expression levels of mRNA of hypoxia genes of gibel carp fed graded dietary of taurine and vitamin C in normoxia for 8 weeks and hypoxia for 12 h. Data with different superscript letters are significantly different (*p* < 0.05); the hypoxic data were labeled with an upper case “AB”, “*” indicates a significant difference (*p* < 0.05) and “**” indicates a significant difference (*p* < 0.01) between values of 0.8 mg/L and 6.5 mg/L dissolved oxygen as determined by *t*-test. (**A**) *hif-1α*, hypoxia-inducible factor 1-α; (**B**) *vegf*, vascular endothelial growth factor; (**C**) *epo*, erythropoietin; (**D**) *ho-1*, heme oxygenase-1; (**E**) *angpt1*, angiopoietin-1; (**F**) *et1*, endothelin 1; (**G**) *nos*, nitric oxide synthase; (**H**) *tf*, transferrin; (**I**) *tfr1*, transferrin receptor protein 1.

Figure 3 shows the expression levels of hypoxia-related genes in gibel carp liver from each group in normoxic and hypoxic environments. In a normoxic environment, the addition of taurine and taurine with vitamin C to the diet had no significant effect on each gene. In a hypoxic environment, the expression of *vegf*, *epo*, *ho-1*, *angpt1*, *nos*, and *tfr1* was increased by the addition of taurine and taurine with vitamin C. Among them, *epo*, *ho-1*, and *tfr1* were significantly increased (*p* < 0.05), while *hif-1α* showed a decreasing trend in expression levels, but there was no significant difference (*p* > 0.05). There was no significant trend in the expression levels of *et1* and *tf*. The results of the two-way ANOVA showed that *ho-1* was significantly affected by DO, the addition of taurine and taurine with vitamin C, and the interaction of DO and the addition of taurine and taurine with vitamin C. DO significantly affected the expression levels of *hif-1α*, *vegf*, *epo*, *ho-1*, and *et1* (*p* < 0.05). None of the other genes was significantly affected by these three factors.

### 3.7. Survival Rate of Fish and Mitochondrial Number in Gill Cells in Hypoxia

**Figure 4:**
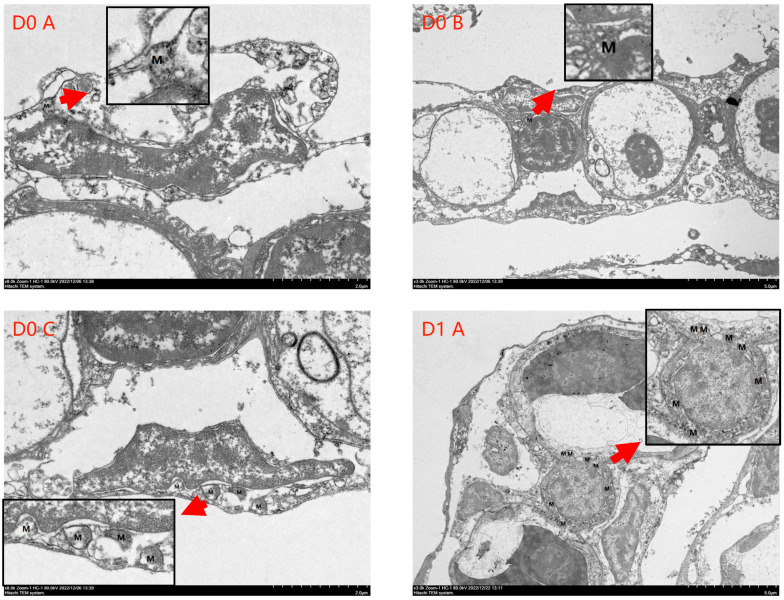

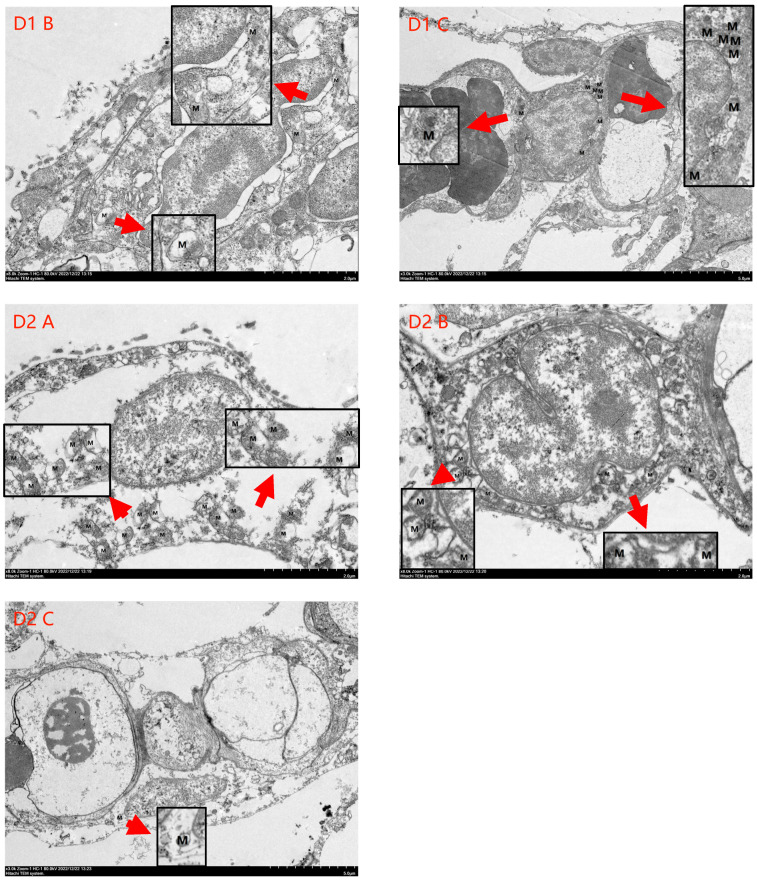
Transmission electron microscopy results of gibel carp gill cells in each group in hypoxia. Note: “D0/D1/D2” denote the three treatment groups, and “A/B/C” represent three samples from the same treatment group. “M” indicates the location and number of mitochondria.

The results of SR and the number of cellular mitochondria (NCM) of the gibel carp in each group under hypoxia are shown in Table 6. With the addition of taurine and taurine with vitamin C, the SR and NCM increased, and the SR of the D2 group was much better than that of the other groups, although the difference was not significant. The statistical results of the NCM for each group are presented in Table 6 and Figure 4. The NCM of the D1 and D2 groups was more than the D0 group; however, the difference was not significant.

## 4. Discussion

### 4.1. Growth Performance and Whole-Body Composition

The results of the present study showed that the addition of taurine and taurine with vitamin C was beneficial to the growth performance of gibel carp, suggesting that taurine and vitamin C play an important role in regulating the growth performance of fish. Numerous studies on other fish species have also reported similar results. For example, the addition of adequate amounts of taurine in the diet has a positive effect on the growth of yellowtail (*Seriola quinqueradiata*) [41], Japanese flounder (*Paralichthys olivaceus*) [42], and turbot [43] by increasing their FBW and SGR and decreasing FCR. Adequate amounts of vitamin C supplements can improve various growth indices, such as WGR and SGR, of Lemeduk fish larvae (*Barbonymus schwanenfeldii*) [44] and yellow catfish [25]. One reason is that vitamin C is an essential nutrient for fish survival. It can act as an enzyme cofactor and help fish maintain optimal health and normal metabolic function, which in turn promotes their growth [45,46]. Taurine is a neuronal trophic factor that has a stimulating effect on the olfactory and gustatory senses of aquatic animals in terms of nitrogen content, water solubility, and acidity and can, therefore, be used as an attractant to promote feeding by aquatic animals [47,48]. Due to the effect of taurine to promote fish feeding and vitamin C to promote fish growth, the addition of both taurine and vitamin C to the feed significantly improved the growth performance of gibel carp.

In terms of whole-body composition, the results showed that the addition of taurine with vitamin C to the feed showed a tendency to increase the crude protein content and decrease the crude lipid content of the gibel carp, but the degree of increase and decrease was not significant compared to the D0 group. Studies have shown that the addition of taurine to feed can increase crude protein content and decrease the crude lipid content of juvenile Atlantic salmon (*Salmo salar*) [49]. The addition of adequate amounts of vitamin C can lead to a significant increase in crude protein and crude lipid content of the fish body [24,50]. However, vitamin C did not significantly affect the body composition of sabaki tilapia (*Oreochromis spilurus*) [51]; therefore, the effect of vitamin C on body composition may depend to some extent on the fish species. The results of this study indicate that exogenous taurine promotes the involvement of sulfur-containing amino acids in protein anabolism by reducing taurine synthesis in fish [52]. At the same time, taurine may be involved in the synthesis of bile acids, promote lipid metabolism [12], increase the activity of the rate-limiting enzyme, and lead to lipid-lowering effects [53]. This effect can be further enhanced by using vitamin C as a coenzyme [45]. The fact that the increase in crude protein and the decrease in crude lipid were not significant may be due to the small amount of added taurine and vitamin C.

### 4.2. Antioxidant Capacity of the Liver (Nrf2 Signaling Pathway)

In the normoxic experiment, the addition of taurine and taurine with vitamin C to the feed significantly increased the enzyme activities of CAT, SOD, T-AOC, and GSH. This was consistent with the results of studies on juvenile black carp [13] and yellowfin bream (*Acanthopagrus latus*) [54], in which the addition of moderate amounts of taurine to the feeds significantly increased the activities of antioxidant enzymes, such as SOD and CAT in fish. Similarly, the addition of vitamin C to feed could also increase the activities of related antioxidant enzymes in yellow catfish [25] and heterozygous gibel carp [28]. It has been suggested that the addition of taurine and taurine with vitamin C could improve the antioxidant ability of gibel carp by promoting the activities of related enzymes [55,56]. Interestingly, the GSH-Px results showed a significant decrease. A rational explanation for this phenomenon is that SOD can decompose the superoxide anion into H_2_O_2_; whereas, both CAT and GSH-Px are important detoxification enzymes for H_2_O_2_ [57]. When the H_2_O_2_ concentration is low, organic peroxides are the preferred substrates for GSH-Px; however, at higher H_2_O_2_ concentrations, they are metabolized by CAT [58]. The addition of taurine and taurine with vitamin C to the diet increased the expression of *nrf2*, *sod*, and the activity of GSH, while the activity of CAT and T-AOC decreased under hypoxic conditions. Compared with the normoxic results, GSH-Px activity was significantly higher in the group with 0.1% taurine + 0% vitamin C (D1) and in the group with 0.1% taurine + 0.1% vitamin C (D2) under hypoxia, which is also consistent with the above explanation. Moreover, the MDA levels of all groups were significantly lower under hypoxia than under normoxia, suggesting that the addition of taurine and vitamin C could lower the MDA levels and alleviate the hypoxic stress of gibel carp by increasing the antioxidant enzyme activities in the hypoxic environment. It can be concluded that the addition of taurine and taurine with vitamin C to the diet can increase the antioxidant capacity of gibel carp in both normoxic and hypoxic environments and alleviate hypoxic stress.

### 4.3. Immune Response of the Liver (NF-κB Signaling Pathway)

Studies have shown that the nuclear transcription factor κB (NF-κB) signaling pathway regulates various physiological activities and that activation of this pathway may promote inflammatory responses and is involved in immune responses [59,60]. Taurine and vitamin C have also been shown to be effective modulators of the organismal inflammatory response and are involved in physiological activities, such as immunomodulation [61,62]. For example, taurine and vitamin C can be used as effective additives to improve the immunity of the olive flounder (*Paralichthys olivaceus*) [63] and rainbow trout [22] by modulating inflammatory cytokines. In this experiment, the relative expression levels of the *il-6* gene in the 0.1% taurine + 0% vitamin C group (D1) and the 0.1% taurine + 0.1% vitamin C group (D2) were significantly lower than those in the 0% taurine + 0% vitamin C group (D0) in normoxia. *Il-6* is a pleiotropic immunocytokine with both pro-inflammatory and anti-inflammatory effects. Studies have shown that prolonged activation of the il-6 signaling pathway can cause major damage to the liver [64]. The levels of other pro-inflammatory cytokines, including *il-8* and *tnf-α*, also showed a decreasing trend. Therefore, the addition of taurine and taurine with vitamin C to the diet can improve the immunity of gibel carp in normoxia by downregulating the expression of pro-inflammatory cytokines. Hypoxic stress can inhibit and impair immune functions in fish [65,66]. In this study, the relative expression levels of *il-10*, *il-6*, and *il-8* were significantly higher under hypoxic conditions than under normoxia, suggesting that hypoxic stress exacerbates the inflammatory response in the liver. The relative expression of *nf-κb* in the 0.1% taurine + 0% vitamin C group (D1), and the 0.1% taurine + 0.1% vitamin C group (D2) was significantly higher than that in the 0% taurine + 0% vitamin C group (D0). *Il-10* also increased, and the level in the D2 group was significantly higher than that in the D0 group. The phenomenon that both anti-inflammatory and pro-inflammatory cytokines show the same expression trend has also been observed in other studies [33,67]. It is speculated that there is a negative feedback mechanism in the NF-κB signaling pathway and that the hypoxic environment promotes the inflammatory response in the liver of gibel carp, while taurine and vitamin C activate the expression of *il-10* by upregulating the expression of *nf-κb* to alleviate hypoxic stress.

### 4.4. Hypoxia Signaling Pathway (HIF-1 Signaling Pathway)

It is well known that hypoxia can be extremely detrimental to the survival and growth of fish [4,5]. Several biological mechanisms resist hypoxic stress in fish. The HIF-1 signaling pathway, which is mainly regulated by hypoxia-inducible factor 1-α (*hif-1α*), plays a crucial role in fish adaptation to hypoxia [68]. In this signaling pathway, *hif-1α*, as an upstream gene, regulates the expression of a variety of genes that can respond to the hypoxic environment and thus control the adaptation of fish to hypoxia [69,70,71,72]. In this experiment, the addition of taurine and taurine with vitamin C to the feed did not significantly affect the relative expression levels of genes in the HIF-1 signaling pathway when the gibel carp were in normoxia. However, compared to normoxia, this pathway was activated during hypoxia, and the expression of the genes *hif-1α*, erythropoietin (*epo*), vascular endothelial growth factor (*vegf*), and heme oxygenase-1 (*ho-1*), which are associated with the production and degradation of erythrocytes, vessels, and hemoglobin [69,70,71], was significantly increased, indicating that the gibel carp was affected by hypoxic stress and activated the corresponding defense mechanisms. At the same time, the addition of taurine and taurine with vitamin C significantly increased the relative expression levels of *epo*, *ho-1*, and *tfr1* under hypoxia. *Tfr1* is the major regulatory receptor in the process of iron uptake in cells and has a crucial function in the development of many diseases [72], suggesting that the defenses of gibel carp against hypoxia were further enhanced. This finding is consistent with previous research, showing that taurine and vitamin C can inhibit the transcription and expression of *hif-1* and, thus, have a therapeutic effect on hypoxia-induced vascular remodeling [73,74]. Under hypoxia, *hif-1α* had a certain decreasing trend between the groups of gibel carp, but the difference was not significant, which was probably the reason for the low dose of added taurine and vitamin C. However, due to the sensitive regulatory effect of *hif-1α* on downstream genes, some genes showed significant differences. The above results suggest that the addition of taurine and taurine with vitamin C may further increase the tolerance of gibel carp to hypoxia by modulating the HIF-1 signaling pathway.

### 4.5. Survival Rate and Mitochondrial Number in Gill Cells of Gibel Carp in Hypoxia

The survival of fish in a hypoxic environment can be used as a visual indicator to assess the damage caused by hypoxia and their ability to tolerate it. In this experiment, although the difference in SR between the groups was not significant, the SR of the 0.1% taurine + 0.1% vitamin C group (D2) was much better than that of the D0 group and the 0.1% taurine + 0% vitamin C group (D1), which is consistent with previous studies showing that taurine can significantly prolong the survival time of hypoxic mice [75] and that vitamin C can improve the hypoxia tolerance of channel catfish (*Ictalurus punctatus*) to a certain extent [76]. At the same time, the results of this experiment indicate that feeding taurine and taurine with vitamin C can improve the antioxidant capacity, immune response, and cardiovascular oxygen transport capacity of gibel carp by modulating the Nrf2, NF-κB, and HIF-1 signaling pathways, thus intuitively showing stronger hypoxia tolerance ability and improved SR. In addition, the results of the swimming ability test conducted at the end of the feeding experiment showed that the simultaneous addition of taurine and vitamin C to the diet could significantly increase the SST of gibel carp in a certain water current environment. The results of previous studies suggested that swimming ability can be used as an important indicator to evaluate the ability of fish to adapt to the environment and that fish with strong swimming ability tend to have better metabolism and a stronger body [77,78,79]. This result can, therefore, be taken as evidence that the addition of taurine and vitamin C to the feed can improve the SR of gibel carp in hypoxic environments by increasing their physical fitness.

Mitochondria are sensitive organelles in cells under stress and are one of the most important organelles that serve as sources of energy for cellular and oxygen metabolism and play important roles in physiological and pathological processes such as cell growth, apoptosis, and senescence [80,81]. Gills are an important organ for oxygen respiration in fish, and their physicochemical state may better reflect the ability of fish to cope with hypoxia [82]. In this experiment, the number of mitochondria in the gill cells of gibel carp was higher in both the group with 0.1% taurine + 0% vitamin C (D1) and the group with 0.1% taurine + 0.1% vitamin C (D2) than in the group with 0% taurine + 0% vitamin C (D0) under hypoxic condition, although the difference was not significant. This result is generally consistent with previous studies showing that both taurine and vitamin C play a crucial role in regulating the functional expression of mitochondria [83,84]. It has been shown that hypoxia leads to high ROS production, resulting in a reduced mitochondrial inner membrane area and decreased oxidative capacity, while mitochondria themselves produce ROS through the respiratory chain, such that large amounts of ROS trigger mitochondrial autophagy in response to a hypoxic environment [85]. In conjunction with other results from this study and other studies, taurine and vitamin C may increase the antioxidant capacity of fish and reduce the damage to organisms by ROS, thereby reducing mitochondrial autophagy and improving mitochondrial function, which in turn may improve the ability of gibel carp to tolerate hypoxia.

## 5. Conclusions

In summary, what we have demonstrated by conducting culture trials as well as hypoxic stress experiments is that the addition of 0.1% taurine + 0.1% vitamin C to the diet could improve the growth performance and swimming ability of gibel carp, while the addition of 0.1% taurine and 0.1% taurine + 0.1% vitamin C improved the antioxidant capacity, immune response, and hypoxia tolerance of gibel carp by modulating the Nrf2, NF-κB, and HIF-1 signaling pathways.

The results of this research confirm that taurine and vitamin C are beneficial feed additives and provide referable nutritional approaches to improve the efficiency of fish farming such as gibel carp and to reduce the damage caused by hypoxic environmental stress in aquaculture. The effects of hypoxia on aquaculture are objective and long term. In the future, more relevant research needs to be invested in alleviating the problem of hypoxia in fish, such as researching more fish species, selecting more suitable feed additives, and determining the optimal amount of additives by setting different additivity levels, in order to ensure the healthy development of the aquaculture industry.

## Figures and Tables

**Table 1:** Ingredient and nutrient composition of the normal diet (% dry matter).

Ingredients	
Fish meal ^1^	14.00
Chicken meal	4.00
Soybean meal ^1^	22.00
Cottonseed meal	5.00
Rapeseed meal ^1^	22.00
Wheat flour ^1^	14.15
Rice bran	10.00
Soybean oil	4.00
Monocalcium phosphate	2.00
Vitamin premix ^2^	0.20
Mineral premix ^3^	2.00
Lysine	0.30
Methionine	0.10
Vc phosphate	0.05
Choline chloride	0.20
Analyzed proximate composition	
Crude protein (%)	39.43 ± 0.43
Crude lipid (%)	7.08 ± 0.33
Crude ash (%)	9.98 ± 0.25

^1^ Fish meal, obtained from Wuxi Tongwei Feedstuffs Co., Ltd., Wuxi, China, crude protein 65.6%, crude lipid 9.5%; soybean meal obtained from Wuxi Tongwei Feedstuffs Co., Ltd., Wuxi, China, crude protein 46.0%, crude lipid 4.3%; rapeseed meal obtained from Wuxi Tongwei Feedstuffs Co., Ltd., Wuxi, China, crude protein 39.2%, crude lipid 6.1%; wheat flour obtained from Wuxi Tongwei Feedstuffs Co., Ltd., Wuxi, China, crude protein 13.1%, crude lipid 4.0%. ^2^ Vitamin premix (IU or mg/kg of premix, purchased by HANOVE Biotechnology Co., Ltd., Wuxi, China): vitamin A, 800,000 IU; vitamin D3, 250,000 IU; vitamin E, 4500 IU; vitamin K3, 600 mg; thiamin, 800 mg; riboflavin, 800 mg; calcium pantothenate, 2000 mg; pyridoxine HCl, 2500 mg; cyanocobalamin, 8 mg; biotin, 16 mg; folic acid, 400 mg; niacin, 2800 mg; inositol, 10,000 mg; vitamin C, 10,000 mg. ^3^ Mineral premix (g/kg of premix, purchased by HANOVE Biotechnology Co., Ltd. Wuxi, China): magnesium sulfate, 1.5%; ferrous sulfate, 30 g; zinc sulfate, 13.5 g; cupric sulfate, 0.8 g; manganese sulfate, 6 g; zeolite was used as a carrier.

**Table 2:** Liver parameters related to antioxidant capacity.

Index	Measurement Methods	Note
CAT	Ammonium molybdenum acid method	Assay kits purchased from Jian Cheng Bioengineering Institute (Nanjing, China); Spectrophotometer (Thermo Fisher Multiskan GO, Shanghai, China).
T-AOC	ABTS method	
SOD	WST-1 method	
MDA	TBA method	
GSH	Microplate method	
GSH-Px	Colorimetric method	

**Table 3:** Primer sequences for RT-qPCR analysis.

Genes	Forward (5′–3′)	Reverse (5′–3′)	Primer Source
*il-10*	AGTGAGACTGAAGGAGCTCCG	TGGCAGAATGGTGTCCAAGTA	[37]
*tgf-β*	GTTGGCGTAATAACCAGAAGG	AACAGAACAAGTTTGTACCGATAAG	[38]
*il-1β*	GCGCTGCTCAACTTCATCTTG	GTGACACATTAAGCGGCTTCA C	[38]
*il-6*	CGGAGGGGCTTAACAGGATG	GCTGGCTCAGGAATGGGTAT	DQ861993.1
*il-8*	ATTGGTGAAGGAATGAGTCT	CCACAGATGACCTTGACAT	KC184490.1
*tnf-α*	CATTCCTACGGATGGCATTTACTT	CCTCAGGAATGTCAGTCTTGCAT	[38]
*nf-κb*	GCTCTGACTGCGGTCTTATAC	GCGCTTCATCGAGGATAGTT	[39]
*cat*	TGAAGTTCTACACCGATGAG	CTGAGAGTGGACGAAGGA	XM_026238665.1
*sod*	TCGGAGACCTTGGTAATGT	CGCCTTCTCATGGATCAC	JQ776518.1
*gpx*	GAAGTGAACGGTGTGAACGC	GATCCCCCATCAAGGACACG	DQ983598.1
*keap1*	CTCCGCTGAATGCTACAA	GGTCATAACACTCCACACT	XM_026245355.1
*nrf2*	TACCAAAGACAAGCAGAAGAAACG	GCCTCGTTGAGCTGGTGTTTGG	[40]
*hif-1α*	CTGCCGATCAGTCTGTCTCC	TTTGTGGAGTCTGGACCACG	DQ306727.1
*vegf*	ATCGAGCACACGTACATCCC	CCTTTGGCCTGCATTCACAC	NM_131408.3
*epo*	CGAAGTGTCAGCATACCGGA	GCAGATGACGCACTTTTCCC	KC460317.1
*ho-1*	GCAAACCAAGAGAAGCCACC	GGAAGTAGACGGGCTGAACC	KC758864
*angpt1*	CCAAACCTCACCAAGCAAGC	GGATTACAGTCCAGCCTCCG	XM_059556208.1
*et1*	TAAAGCAGCGTCAGACAGGG	CTGCCAGCTTGTGTTTGCAT	NM_131519.1
*nos*	GGGGACCCTCCTGAAAATGG	TTCTGTCCTCAACGCTGGTG	AY644726.1
*tf*	CCGAGAAGATGCACGCAAAG	TGTGCATGCCTTGACCAGAT	AF518747.1
*tfr1*	CTTTGTCAACGAAGTGGCTGAAT	TACCAAAGAAAATGTGGCGGAAC	XM_052542523.1
*β-actin*	TCCATTGTTGGACGACCCAG	TGGGCCTCATCTCCCACATA	LC382464.1

Note: *il-10*, interleukin-10; *tgf-β*, transforming growth factor-β; *il-1β*, interleukin-1β; *il-6*, inter leukin-6; *il-8*, interleukin-8; *tnf-α*, tumor necrosis factor-α; *nf-κb*, nuclear factor kappa-β; *cat*, catalase; *sod*, superoxide dismutase; *gpx*, glutathione peroxidase; *keap1*, recombinant kelch-like ECH-associated protein 1; *nrf2*, nuclear factor erythroid 2-related factor 2; *hif-1α*, hypoxia-inducible factor 1-α; *vegf*, vascular endothelial growth factor; *epo*, erythropoietin; *ho-1*, heme oxygenase-1; *angpt1*, angiopoietin-1; *et1*, endothelin 1; *nos*, nitric oxide synthase; *tf*, transferrin; *tfr1*, transferrin receptor protein 1; *β-actin*, beta-actin.

**Table 4:** Growth performance, whole-body composition, and swimming ability of gibel carp fed graded dietary of taurine and vitamin C levels for 8 weeks.

Parameters	Dietary Taurine and Vitamin C Levels (%)		
	0 + 0	0.1 + 0	0.1 + 0.1
Growth performance			
IBW (g) ^1^	41.92 ± 0.08	41.82 ± 0.04	41.78 ± 0.11
FBW (g) ^2^	101.57 ± 0.58	102.97 ± 0.84	104.20 ± 0.77
FCR ^3^	1.37 ± 0.02	1.39 ± 0.02	1.43 ± 0.01
SGR (% day-1) ^4^	0.95 ± 0.01 ^b^	0.97 ± 0.01 ^ab^	0.98 ± 0.01 ^a^
WGR (%) ^5^	142 ± 0.02 ^b^	146 ± 0.02 ^ab^	149 ± 0.02 ^a^
SR (%) ^6^	100.0 ± 0.00	100.0 ± 0.00	100.0 ± 0.00
Whole-body composition (%)			
Moisture	75.21 ± 1.02	74.88 ± 0.95	74.99 ± 0.62
Crude protein	15.92 ± 0.38	15.80 ± 0.42	16.42 ± 0.36
Crude lipid	2.62 ± 0.93	2.37 ± 0.75	1.82 ± 0.26
Ash	4.56 ± 0.16	4.75 ± 0.06	4.76 ± 0.14
Swimming ability			
SST (sec) ^7^	37.17 ± 8.63 ^b^	43.00 ± 8.79 ^b^	142.20 ± 8.63 ^a^

Note: Data are mean value ± SEM. Means in the same row with different super scripts are significantly different (*p* < 0.05). ^1^ IBM, Initial body weight. ^2^ FBW, Final body weight. ^3^ FCR, Feed conversion ratio = dry feed fed (g)/wet weight gain (g). ^4^ SGR, Specific growth rate (%/d) = 100 × [(Ln (final body weight (g)) − Ln (initial body weight (g)))/days]. ^5^ WGR, Weight gain rate (%) = 100 × (final weight (g) − initial weight (g))/initial weight (g). ^6^ SR, Survival rate (%) = 100 × (survival fish number/total fish). ^7^ SST, Sustained swimming time (s).

**Table 5:** Antioxidant enzyme activities and MDA of gibel carp fed graded dietary of taurine and vitamin C levels in normoxia for 8 weeks and hypoxia for 12 h.

Experimental Groups	Parameters					
Dissolved Oxygen (mg/L)/Taurine and Vitamin C Levels (%)	CAT (U/mgprot) ^1^	T-AOC (mmol/gprot) ^2^	SOD (U/mgprot) ^3^	MDA (nmol/mgprot) ^4^	GSH (μmol/gprot) ^5^	GSH-Px (U/mgprot) ^6^
6.5/(0 + 0)	235.26 ± 6.40 ^c^	0.20 ± 0.03 ^b^	9.72 ± 0.41 ^b^	8.12 ± 0.43	341.36 ± 39.41 ^b^	389.08 ± 24.74 ^a^
6.5/(0.1 + 0)	256.69 ± 4.69 ^b^	0.47 ± 0.06 ^a^	11.29 ± 0.28 ^a^	6.95 ± 0.24	492.17 ± 16.72 ^a^	344.99 ± 29.15 ^ab^
6.5/(0.1 + 0.1)	287.47 ± 2.43 ^a^	0.34 ± 0.05 ^ab^	11.07 ± 0.30 ^a^	7.13 ± 0.67	416.84 ± 39.82 ^ab^	299.65 ± 8.92 ^b^
0.8/(0 + 0)	307.08 ± 10.93 ^A^	0.39 ± 0.04 ^A^	7.90 ± 0.53	3.17 ± 0.41	355.38 ± 26.57 ^B^	448.10 ± 31.79
0.8/(0.1 + 0)	258.22 ± 14.23 ^B^	0.46 ± 0.06 ^A^	7.42 ± 0.53	2.70 ± 0.43	476.97 ± 41.14 ^A^	529.97 ± 28.06
0.8/(0.1 + 0.1)	255.88 ± 13.15 ^B^	0.19 ± 0.02 ^B^	6.76 ± 0.19	3.48 ± 0.26	377.81 ± 21.70 ^B^	483.13 ± 15.06
Dissolved oxygen (mg/L)						
6.5	261.54 ± 6.14	0.34 ± 0.04	10.75 ± 0.24 ^y^	7.34 ± 0.27 ^y^	416.79 ± 25.22	344.54 ± 15.55 ^x^
0.8	272.53 ± 9.63	0.35 ± 0.04	7.33 ± 0.26 ^x^	3.10 ± 0.22 ^x^	401.79 ± 20.87	487.34 ± 17.47 ^y^
Taurine and vitamin C levels (%)						
0 + 0	267.18 ± 13.80	0.32 ± 0.04 ^b^	8.82 ± 0.44	5.15 ± 0.86	349.15 ± 21.47 ^b^	418.59 ± 21.39
0.1 + 0	257.45 ± 7.07	0.46 ± 0.04 ^a^	9.35 ± 0.65	4.83 ± 0.68	483.72 ± 22.89 ^a^	447.75 ± 37.64
0.1 + 0.1	274.83 ± 7.19	0.26 ± 0.03 ^b^	8.92 ± 0.67	5.11 ± 0.71	395.55 ± 21.32 ^b^	381.20 ± 33.15
Two-way ANOVA						
Dissolved oxygen	0.073	0.725	0.000	0.000	0.619	0.009
Taurine and vitamin C levels	0.219	0.000	0.341	0.138	0.002	0.210
Interaction	0.000	0.006	0.009	0.331	0.714	0.025

Note: Data are mean value ± SEM. Means with different superscript letters in the same column are significantly different (*p* < 0.05), normoxic data are labeled with a lower case “abc” and hypoxic data are labeled with an upper case “AB”, and the superscript letter “x” and “y” indicates a significant difference (*p* < 0.05) between values of 0.8 mg/L and 6.5 mg/L dissolved oxygen as determined by *t*-test. Means with the same letters or no letters indicate no significant difference among treatments. ^1^ CAT, catalase; ^2^ T-AOC, total antioxidant capacity; ^3^ SOD, superoxide dismutase; ^4^ MDA, malondialdehyde; ^5^ GSH, glutathione; ^6^ GSH-Px, glutathione peroxidase.

**Table 6:** Survival rate of fish and the number of cellular mitochondria of gibel carp in hypoxia.

Parameters	Dietary Taurine and Vitamin C levels (%)		
	0 + 0	0.1 + 0	0.1 + 0.1
SR (%) ^1^	12.50 ± 12.50 ^a^	8.33 ± 4.17 ^a^	33.33 ± 4.17 ^a^
NCM (pcs) ^2^	2.00 ± 1.00 ^a^	6.00 ± 1.53 ^a^	5.67 ± 2.91 ^a^

Note: Data are mean value ± SEM. Means in the same row with different superscripts are significantly different (*p* < 0.05). ^1^ SR, survival rate (%) = 100 × (survival fish number/total fish). ^2^ NCM (pcs), The number of cellular mitochondria.

## Data Availability

Data are contained within the article.
